# Supplementary material for: EIF4EBP1 is transcriptionally upregulated by MYCN and associates with poor prognosis in neuroblastoma
Source: Cell Death Discov. 2022 Apr 4;8:157. doi: 10.1038/s41420-022-00963-0 (PMC8980029; doi:10.1038/s41420-022-00963-0)
Supplement: Supplementary file 1 — Supplementary figure legends [file 41420_2022_963_MOESM1_ESM.docx]

**SUPPLEMENTARY FIGURE LEGENDS**

**Supplementary Figure 1:** *EIF4EBP1* mRNA expression correlates with event-free survival in NB patients.

(a-b) Kaplan-Meier survival estimates of event-free survival of NB patients stratified by their *EIF4EBP1* mRNA expression levels (median cut off) in the SEQC (a), Kocak (b) and NRC (c) cohorts. (d-h) Kaplan-Meier estimates of event-free survival of patients with *MYCN*-non-amplified NB (d, e), high-risk NB (f) or stage 4 NB (g, h) stratified by their *EIF4EBP1* mRNA expression levels in the indicated NB cohorts. Significance was determined by log rank test. Data were obtained from the R2: Genomics Analysis and Visualization Platform.
