# Supplementary figures and images for: EIF4EBP1 is transcriptionally upregulated by MYCN and associates with poor prognosis in neuroblastoma
Source: Cell Death Discov. 2022 Apr 4;8:157. doi: 10.1038/s41420-022-00963-0 (PMC8980029; doi:10.1038/s41420-022-00963-0)

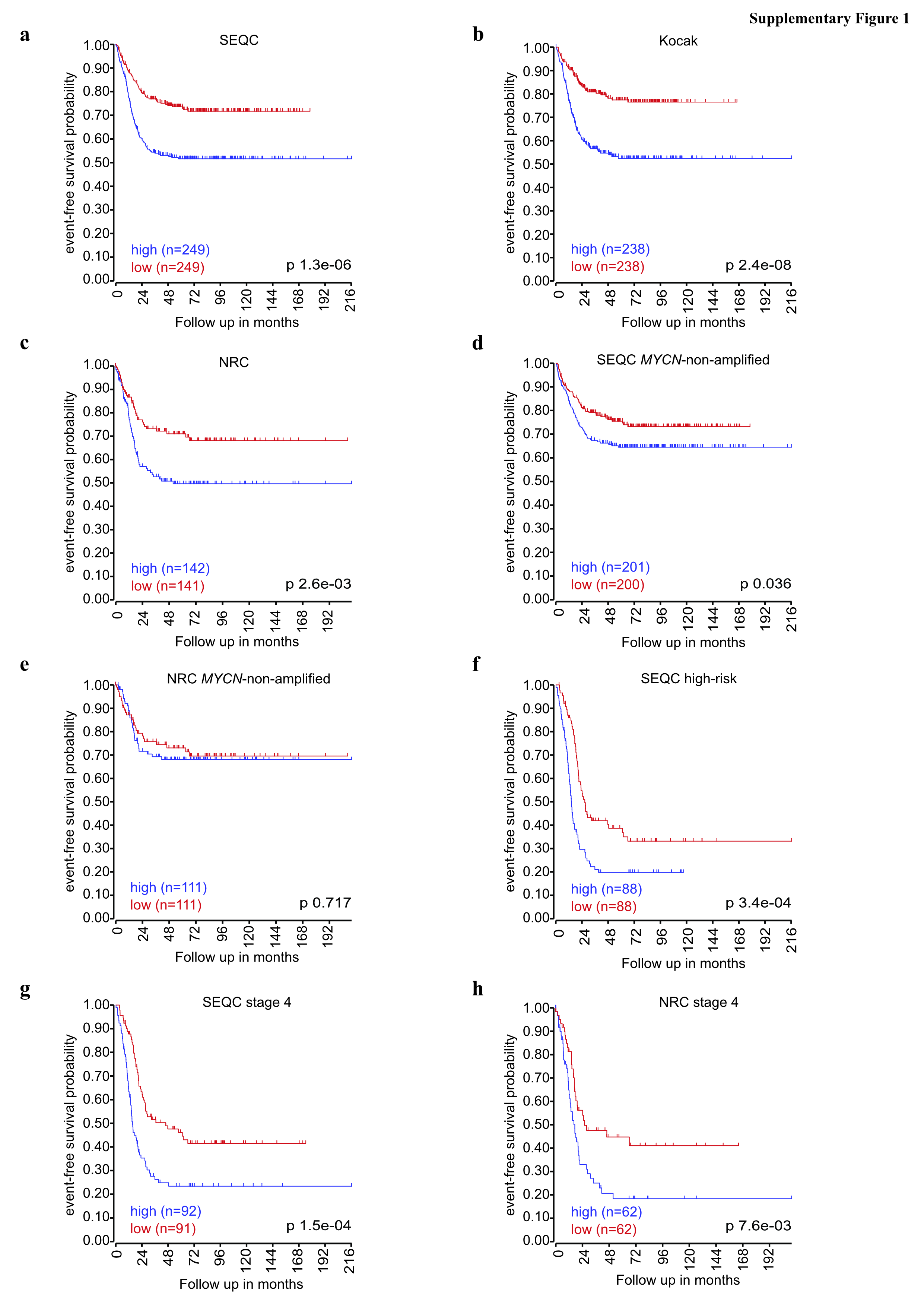

Supplement: Supplementary file 2 — Supplementary Figure 1 [file 41420_2022_963_MOESM2_ESM.tif]
