# Supplementary material for: EIF4EBP1 is transcriptionally upregulated by MYCN and associates with poor prognosis in neuroblastoma
Source: Cell Death Discov. 2022 Apr 4;8:157. doi: 10.1038/s41420-022-00963-0 (PMC8980029; doi:10.1038/s41420-022-00963-0)
Supplement: Supplementary file 3 — Supplementray Table 1 [file 41420_2022_963_MOESM3_ESM.docx]

**Supplementary table 1: Primer list**

| **Target gene** | **Forward** | **Reverse** |
| --- | --- | --- |
| ***eIF4EBP1*** | AGCCCTTCCAGTGATGAGC | TGTCCATCTCAAACTGTGACTCTT |
| ***MYCN*** | TGAGCGATTCAGATGATGAAGA | GCATCGTTTGAGGATCAGC |
| ***GUSB*** | GTTTTTGATCCAGACCCAGATG | GCCCATTATTCAGAGCGAGTA |
| ***PPIA*** | TTATTTGGGTTGCTCCCTTC | AAGTGTGCCAAATCTGCAAG |
